# Efficacy of transcranial direct current stimulation in reducing impulsivity in borderline personality disorder (TIMBER): study protocol of a randomized controlled clinical trial

**DOI:** 10.1186/s13063-019-3427-z

**Published:** 2019-06-10

**Authors:** Juliana Teti Mayer, Magali Nicolier, Damien Gabriel, Caroline Masse, Julie Giustiniani, Charline Compagne, Pierre Vandel, Lionel Pazart, Emmanuel Haffen, Djamila Bennabi

**Affiliations:** 1 Service de Psychiatrie de l’Adulte, Centre Hospitalier Universitaire de Besançon, 25030 Besançon Cedex, France; 2 Centre d’Investigation Clinique, INSERM CIC 1431, Centre Hospitalier Universitaire de Besançon, 25030 Besançon Cedex, France; 3 Laboratoire de Neurosciences Intégratives et Cliniques EA, Université de Bourgogne Franche-Comté, 19 rue Ambroise Paré, 25000 Besançon, France; 4 Centre Expert Dépression Résistante FondaMental, Centre Hospitalier Universitaire de Besançon, 25030 Besançon Cedex, France; 5 Centre Mémoire Ressources et Recherche, Centre Hospitalier Universitaire de Besançon, 25030 Besançon Cedex, France

**Keywords:** Borderline personality disorder, Transcranial direct current stimulation, Impulsivity, Risk-taking, Dorsolateral prefrontal cortex

## Abstract

**Background:**

Impulsivity is a core feature of borderline personality disorder (BPD) and is closely related to suicide risk and destructive and aggressive behaviors. Although transcranial direct current stimulation (tDCS) has shown its promising effects as an intervention to modulate impulsivity, no study has explored its potential regarding BPD.

**Methods/design:**

This is a multicenter, crossover, double-blind study comparing active versus sham tDCS (2 mA, 30 min), applied over the dorsolateral prefrontal cortex for five consecutive days in 50 BPD patients. Participants will be assessed for impulsivity, depressive symptoms, and suicide risk. The main efficacy criteria on reduction of impulsivity will be the amplitude variation of one specific evoked potential detected by electroencephalography (EEG) during the balloon analogue risk task. Baseline measures will be compared to scores obtained immediately after sessions, then 12 and 30 days later.

**Discussion:**

This study investigates the safety and effects of tDCS, which may have a significant impact on impulsivity in patients with BPD and may be useful to reduce risky behaviors.

**Trial registration:**

ClinicalTrials.gov, NCT03498937. Registered on 17 April 2018.

**Electronic supplementary material:**

The online version of this article (10.1186/s13063-019-3427-z) contains supplementary material, which is available to authorized users.

## Background

Borderline personality disorder (BPD) is characterized by a pervasive pattern of instability, affecting impulse control, emotional regulation, cognitive processing, and interpersonal relationships [1]. It is the most frequent personality disorder, with a prevalence of 1.6% in the general population and 15 to 50% in the psychiatric inpatient population [17]. Suicide is one of the leading causes of death in this population, with a rate far greater than that in the general population, estimated to be between 8 and 11% [12, 16]. In BPD, impulsivity has been shown to be closely linked to suicide risk and destructive and aggressive behavior, and it has been related to poor treatment program adherence and intense healthcare use ([2, 13, 18–20]).

Impulsivity is considered to involve failure of inhibitory control, either motor or cognitive, and deficits of the reward valuation system. From a neurobiological perspective, the prefrontal cortex is considered a critical region in the cognitive control of behaviors. Previous studies have associated hypoactivation of the dorsolateral prefrontal cortex (dlPFC) and the dorsal part of the anterior cingulate cortex to impulsivity measures in BPD [5, 15].

Despite its clinical significance, BPD remains a challenge to treat and manage. Pharmacological treatments have shown some promising effects with regard to impulsive behaviors, although results remain equivocal [14]. Efficacy of psychotherapies on BPD has been recently reviewed [4], with most trials focusing on dialectical behavior therapy and psychodynamic therapies. However, effect sizes were small and follow-up results were unstable, with no evidence of increase in treatment retention. Therefore, there is a clear need for alternative interventions that target both the cognitive control issues associated with impulsivity as well as its underlying neural dysfunction.

Transcranial direct current stimulation (tDCS) is a promising, low-risk, non-invasive neuromodulation technique that relies on the application of a weak direct current of 1–2 mA to generate regional changes in cortical excitability, which, depending on the duration and the polarity, can last for several minutes up to a few hours after stimulation [10, 11]. Due to the key role of the prefrontal cortex in cognitive control, previous studies have applied tDCS over this cortical area in healthy subjects and clinical populations (with a diagnosis of depression, attention deficit/hyperactivity disorder, or substance abuse) and have reported a significant reduction of different aspects of impulsivity, such as inhibitory control, planning, delay-discounting, and risk-taking [3, 6–9]. tDCS over the prefrontal cortex may thus be a valuable therapeutic approach that can modulate impulsivity in BPD, leading to meaningful changes in suicidal and risky behaviors.

Hence, we propose to evaluate, for the first time, the clinical benefits of tDCS on reducing impulsivity in a BPD population. The main objective will be to investigate the efficacy of 1 week of bilateral tDCS (five consecutive twice-daily sessions), applied over the dlPFC of patients with BPD, in reducing impulsivity within the following 3 weeks. This study consists of a multicenter, sham-controlled, randomized crossover double-blinded trial, comparing active tDCS versus sham tDCS. We hypothesize that the active tDCS sessions would induce a greater reduction in impulsive behavior on risk-taking tasks compared to the sham sessions.

## Methods/design

### Study setting and overview

**Fig. 1:**
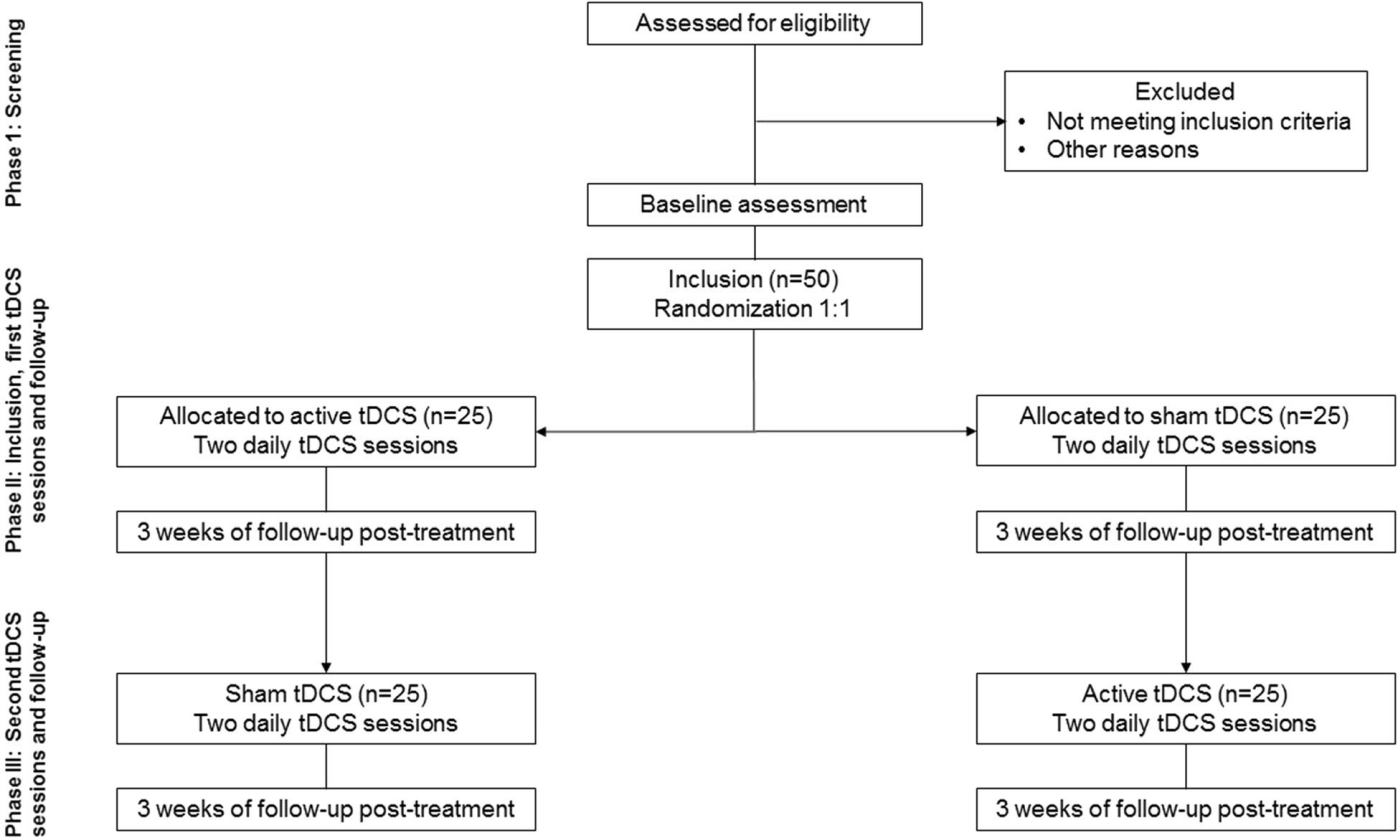
Study flow diagram

The research will be carried out in the psychiatric departments of three clinical centers in France (in the cities of Besançon, Nancy, and Rouffach), aiming to recruit 50 patients with BPD over the course of 2 years. Potential participants will be recruited from inpatient and outpatient services in each center. After they have been provided with a complete description of the study, written informed consent will be obtained. They will be assigned to two groups, and those who start by active stimulation sessions will then be submitted to sham sessions and vice versa (Fig. 1).

Baseline measures will include a clinical assessment of impulsivity, suicidal thoughts, and depression severity based on self-reports and clinician rated scales. These results will be compared to those that will be obtained immediately after the last tDCS session and then 12 and 30 days following the start of the sessions. Active and sham stimulation session outcomes will also be compared. In addition, task-based measures of behavioral and cognitive impulsivity will be administered, and data from electroencephalography (EEG) will be collected during one of the impulsivity tasks, pre- and post-tDCS administration, after 1 and 10 sessions, and 12 and 30 days later (see “Expected outcomes” section below for details).

### Inclusion criteria

Eligible patients will be invited to take part in this trial according to the following criteria: (1) male and female patients over 18 years old; (2) well-established diagnosis of BPD according to the Diagnostic and Statistical Manual of Mental Disorders, fifth edition (DSM-5) [1] and confirmed by the Structured Clinical Interview for DSM-IV-Axis II personality disorders (SCID-II); (3) absence of addictive comorbidities (except tobacco, tea, and coffee) and severe progressive neurologic and/or somatic disease.

### Exclusion criteria

Patients will be excluded if they are identified as having any of the following: (1) younger than 18 years old; (2) severe chronic psychiatric disorders, including schizophrenia, paranoia, or bipolar disorder type I and II; (3) acute serious and/or unstable medical conditions that would compromise the patient’s participation in the study, according to medical judgment; (4) use of antipsychotic and mood stabilizing treatments; (5) contraindications to tDCS, e.g., metallic foreign body in the head or medical devices implanted in the brain; (6) pregnancy; (7) concurrent participation in another trial; (8) no coverage by national health insurance; (9) measure of protection or guardianship of justice.

### Interventions

For tDCS and EEG, a wireless hybrid EEG/tDCS 8-channel neurostimulator system (StarStim®, Neuroelectrics, Barcelona, Spain) that allows for stimulation and EEG recording, with a sham and double-blind mode will be used.

Direct current will be generated by the neurostimulator and transmitted by two saline-soaked synthetic sponge electrodes. Intensity of 2 mA will be induced by two circular carbon rubber core electrodes in saline-soaked surface sponges (25 cm^2^), placed in a neoprene head cap over the dlPFC (anode position over F4 and cathode over F3, according to the EEG 10–20 International System).

In the active group, current will be delivered for 30 min, twice a day, for 5 days consecutively. For sham stimulation the procedure will be identical, except that the current will gradually ramp down to zero after 30 s, thus leading to the same initial sensations of active tDCS.

Electrophysiological signals will be recorded with the same neurostimulator system, with a sampling rate of 500 Hz. Eight dry electrodes will be placed over regions of interest for recording patients’ neuronal activity during an adapted version of the balloon analogue risk task (BART) to EEG.

Antipsychotic and mood-stabilizing treatments are forbidden during the progress of the study due to their negative impact on the action of tDCS by reducing cortical excitability.

### Outcomes

Task-based measures of behavioral and cognitive impulsivity will be administered before and after tDCS or sham stimulation. Additionally, EEG data will be collected during the BART, and resting-state EEG data will be collected pre- and post-tDCS administration to confirm engagement of the targeted brain region and to delineate the neural pathways underlying the effects of tDCS on impulsivity.

Our main efficacy criteria on reduction of impulsivity will be the amplitude variation of one specific evoked potential detected by EEG during the BART: feedback-related negativity (FRN). This reward-prediction error component is more negative when outcomes are worse than expected and more positive when they are better. The amplitude of the FRN will be compared before stimulation sessions and 5, 12, and 30 days after beginning active and/or sham tDCS.

Secondary efficacy criteria are:

- Changes in impulsivity (self-reported) by comparison of scores from the French version of the Barratt Impulsiveness Scale (BIS-10) obtained before the beginning of stimulation sessions and 5, 12, and 30 days after active and/or sham tDCS
- Changes in impulsivity (self-reported) by comparison of scores from the Urgency, Premeditation (lack of), Perseverance (lack of), Sensation Seeking, Positive Urgency Impulsive Behavior Scale (UPPS-P) obtained before the beginning of stimulation sessions and 5, 12, and 30 days after active and/or sham tDCS
- Changes in depression severity (clinician-rated) by comparison of scores from the Hamilton Depression Rating Scale (HDRS) obtained before the beginning of stimulation sessions and 5, 12, and 30 days after active and/or sham tDCS
- Changes in depression severity (clinician-rated) by comparison of scores from the Montgomery and Asberg Depression Rating Scale (MADRS) obtained before the beginning of stimulation sessions and 5, 12, and 30 days after active and/or sham tDCS
- Changes in depression severity (self-reported) by comparison of scores from the 16-item Quick Inventory of Depressive Symptomatology (QIDS-SR16) obtained before the beginning of stimulation sessions and 5, 12, and 30 days after active and/or sham tDCS
- Changes in suicidal thoughts (clinician-rated) by comparison of scores from the Columbia-Suicide Severity Rating Scale (C-SSRS) obtained before the beginning of stimulation sessions and 5, 12, and 30 days after active and/or sham tDCS
- Changes in impulsivity (behavioral assessment of inhibitory control) by comparison of scores from the experimental Go/No-Go and Stroop tasks obtained before the beginning of stimulation sessions and 5, 12, and 30 days after active and/or sham tDCS

### Study procedure

**Fig. 2:**
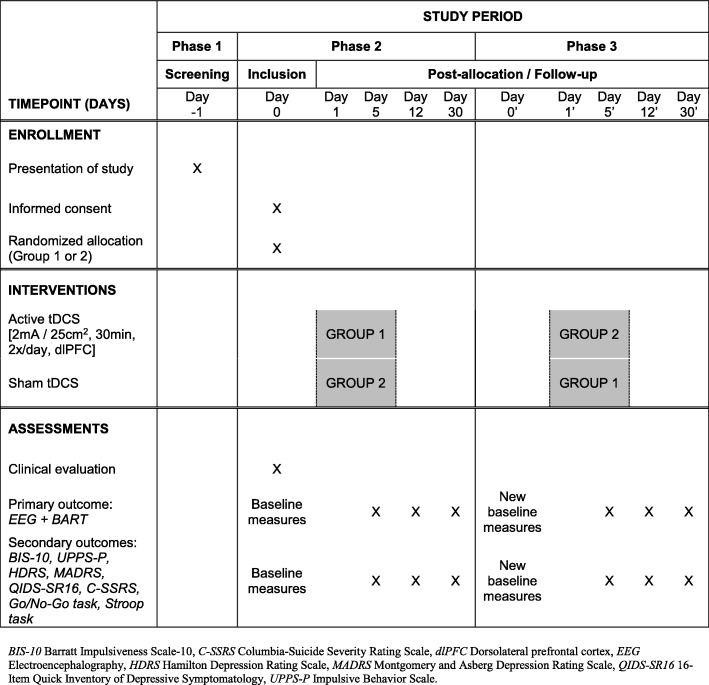
Randomized cross-over design for TIMBER (Standard Protocol Items: Recommendations for Interventional Trials (SPIRIT) figure)

The study will have three phases. During the first phase, subjects will be screened using the inclusion and exclusion criteria. Information on the implementation of the study and the objectives of the research will be given to each subject by trained staff and/or health care providers. Enrollment and tDCS treatment will be scheduled in the following weeks for eligible participants. The detailed procedure is displayed in Fig. 2.

The second phase will correspond to both inclusion of volunteers and the period of the tDCS sessions. All volunteers will have to sign the informed consent. Diagnosis will be established by an experienced psychiatrist, in accordance with DSM-5 criteria and confirmation by the SCID-II, as stated in the inclusion criteria section. Clinical assessment will be performed and behavioral baseline scores will be collected. Following completion of baseline clinical measures, subjects will be randomly assigned using a computer-generated randomization list with the information stored on a centralized computer to receive either active or sham tDCS. Predefined codes assigned to either real or sham stimulation will be used by trained staff and health care professionals to start the stimulator, allowing a double-blind study design. The first tDCS session will be delivered on a Monday. Two daily sessions will be performed during the following days up to Friday. The clinical and behavioral assessment will be performed once the last tDCS session has been delivered (day 5) and then 12 (day 12) and 30 days (day 30) following the start of the sessions.

Following the last assessment, subjects who underwent active stimulation sessions will then be submitted to sham sessions and vice versa (third phase). Then, once again, baseline measures will be performed before new sessions start, followed by evaluation immediately after their end and then 12 and 30 days after the start.

### Sample size

Our primary efficacy outcome concerns changes in impulsivity by means of assessment of risk in conjunction with EEG measures. In this context, a sample size calculation based on an expected difference of 2 mV between the variation of amplitude of the FRN recorded by EEG during the BART, with a standard deviation for paired-differences of 3 and with an autocorrelation of 10% between measures in the same subject, was performed. Considering a significance level of 5%, a power of 90%, and a drop-out rate of 10% of patients, 50 patients should be included to meet the objectives of the study.

### Withdrawals

Subjects are informed before inclusion that they may withdraw their consent for treatment at any time during the study, with no need to justify their decision and without compromising their original follow-up or treatment in their respective service. Patients may as well be removed at any time from the study if one or more of the following is detected:

- Increase in suicide risk: observed in the third item of the HDRS and defined as an increase of 2 points for baseline scores between 0 and 2, and 1 point for a baseline score of 3
- Adverse events
- Any exclusion criteria

In all cases, patients will be conducted to specialized care and appropriate follow-up, granted by the national health insurance coverage. Every subject withdrawal will be registered, reporting motives as detailed as possible.

### Data management and statistical analyses

Collected information will be stored in physical files (Case Report Files (CRFs)), registered under each participant’s randomization code to respect confidentiality at all times. All researchers and trained staff called upon to collaborate in the tests are bound to secrecy. An anonymous electronic database (eCRFs) will be created, with controlled access, on the platform CleanWEB™, allowing multicenter data analysis.

All randomized patients will be used for the efficacy analyses. Qualitative variables will be described in terms of effective, absolute, and relative frequencies for each modality. Quantitative variables will be described in terms of minimum and maximum, quartiles, means, and standard deviations.

Comparison between active and sham stimulation will be based on the Student’s *t*-test for quantitative variables and the Wilcoxon test for semi-quantitative variables. The Student’s *t*-test or the Mann-Whitney test will be used for comparison of amplitude variation detected by the EEG. Analyses will be performed using Stata Software release 10.1 (StataCorp, Collège Station, TX, USA). No interim analyses will be performed.

### Monitoring

A data monitoring committee will be designated by the University Hospital of Besançon, in accordance with French legislation and the European Medicine Agency’s Guideline on Data Monitoring Committee. Monitors will have documented competence to follow up the research and no competing interests. Monitoring visits to all centers will take place annually to verify adequate progress of the research and respect for ethical regulations.

Any adverse events or unexpected outcomes will be reported online as soon as researchers and/or staff become aware of their occurrence. The electronic report will be sent directly to the University Hospital of Besançon, which will be in charge of informing national health surveillance agencies.

### Ethics and dissemination

The study was prospectively registered on ClinicalTrials.org as “Effects of tDCS on impulsiveness among people suffering from Borderline Personality Disorder (TIMBER)”, identifier NCT03498937 (https://clinicaltrials.gov/ct2/show/NCT03498937). This protocol is version 3.0, 23 March 2018, approved by the French Committee for the Protection of Persons Sud-Méditerrannée II, under the number 218 B13. It is written in line with the Principles of Helsinki and adheres to the Standard Protocol Items: Recommendations for Interventional Trials (SPIRIT) guidelines (Additional file 1: SPIRIT Checklist—TIMBER). A record of previous versions of the study protocol is kept, and eventual modifications or corrections will be reported and justified to the University Hospital of Besançon.

Prior to study enrollment, participants will receive a complete description of the study and written informed consent will be obtained from the volunteers, who may withdraw their consent for treatment at any time during the study, with no need to justify their decision and without compromising their original follow-up or treatment.

Data management and monitoring are in line with the French Public Health Code’s guidance on good clinical practice to conduct trials of human participants (especially article L.1121–3), the French Jardé Law (No. 2012–300, from 5 March 2012) and the European “Note for Guidance on Good Clinical Practice” (ICH E6, CPMP/ICH/135/95). Trial results will be disseminated in peer-reviewed publications and clinical/academic conferences. This study is also associated with a research project towards a PhD degree (JTM) at the University of Bourgogne-Franche-Comté.

## Discussion

tDCS is a promising technique that offers the opportunity to modify brain dysfunctions observed in certain psychiatric diseases and to improve their course. This technique is likely to be a therapeutic tool in specific populations characterized by high levels of impulsivity. Moreover, it could constitute an interesting alternative strategy to pharmacological treatment classically used to reduce impulsivity due to its safety, ease of use, and favorable tolerability profile.

To our knowledge, this pilot study will be the first randomized controlled trial to investigate the neuromodulatory effects of tDCS in patients with BPD, with a focus on impulsive behaviors. The results obtained from this study will be valuable in order to establish new concepts for the treatment of BPD. In addition, providing new information regarding the possible effects of tDCS on impulsivity, the combination of EEG measures, and this neuromodulation technique will be useful in the study of neural mechanisms underlying impulsive behavior. The concomitant use of EEG and behavioral measures of impulsivity may allow characterization of behavioral and cognitive aspects of impulsivity in BPD, as well as provide a better insight into cortical brain areas and neural pathways underlying the effects of tDCS on impulsivity.

The study is strengthened by a crossover double-blind approach to tDCS stimulation and the assessment of impulsivity across multiple domains. The bilateral application of tDCS over the dlPFC and the frequency have been chosen in line with studies applying tDCS to reduce impulsivity. Currently in France, tDCS application is limited to the research field and discussion of what may be the optimal electrode configuration, stimulation intensity, and frequency is still ongoing. Hence, further studies are imperative in order to establish the technique as an evidence-based therapeutic tool, including for BPD. The results obtained from this trial will be valuable in order to design larger randomized clinical trials and contribute to a field which has not been widely studied.

## Trial status

The study is recruiting patients from November 2018 until May 2020, aiming to enroll 50 patients.

## Additional file

Additional file 1: SPIRIT Checklist for TIMBER Protocol. (DOCX 55 kb)

## Data Availability

Data sharing is not applicable to this article as datasets were not yet generated during the current study.

## Abbreviations

BART: Balloon analogue risk task
BIS-10: Barratt Impulsiveness Scale-10
BPD: Borderline personality disorder
C-SSRS: Columbia-Suicide Severity Rating Scale
dlPFC: Dorsolateral prefrontal cortex
DSM-5: Diagnostic and Statistical Manual of Mental Disorders, fifth edition
EEG: Electroencephalography
FRN: Feedback-related negativity
HDRS: Hamilton Depression Rating Scale
MADRS: Montgomery and Asberg Depression Rating Scale
QIDS-SR16: 16-Item Quick Inventory of Depressive Symptomatology
SCID-II: Structured Clinical Interview for DSM-IV-Axis II personality disorders
tDCS: Transcranial direct current stimulation
UPPS-P: Impulsive Behavior Scale
